# Factors associated with lifetime suicide attempts in bipolar disorder: results from an Italian nationwide study

**DOI:** 10.1007/s00406-021-01343-y

**Published:** 2021-10-15

**Authors:** Massimiliano Buoli, Bruno Mario Cesana, Simone Bolognesi, Andrea Fagiolini, Umberto Albert, Gabriele Di Salvo, Giuseppe Maina, Andrea de Bartolomeis, Maurizio Pompili, Claudia Palumbo, Emi Bondi, Luca Steardo, Pasquale De Fazio, Mario Amore, Mario Altamura, Antonello Bellomo, Alessandro Bertolino, Marco Di Nicola, Guido Di Sciascio, Andrea Fiorillo, Emilio Sacchetti, Gabriele Sani, Alberto Siracusano, Giorgio Di Lorenzo, Alfonso Tortorella, A. Carlo Altamura, Bernardo Dell’Osso, the ISBD Italian Chapter Epidemiologic Group

**Affiliations:** 1 Department of Neurosciences and Mental Health, Fondazione IRCCS Ca’Granda Ospedale Maggiore Policlinico, Via F. Sforza 35, 20122 Milan, Italy; 2 Department of Pathophysiology and Transplantation, University of Milan, Milan, Italy; 3 Department of Clinical Sciences and Community Health, Unit of Medical Statistics, Biometry and Bioinformatics “Giulio A. Maccacaro”, Faculty of Medicine and Surgery, University of Milan, Milan, Italy; 4 University of Siena School of Medicine, Siena, Tuscany, Italy; 5 Department of Medicine, Surgery and Health Sciences, Department of Mental Health, UCO Clinica Psichiatrica, ASUGI-Azienda Sanitaria Universitaria Giuliano-Isontina, University of Trieste, Trieste, Italy; 6 San Luigi Gonzaga Hospital, University of Turin, Turin, Italy; 7 Laboratory of Molecular Psychiatry and Translational Psychiatry, Unit of Treatment Resistant Psychosis, Section of Psychiatry, Department of Neuroscience, Reproductive Science and Odontostomatology, University School of Medicine of Napoli Federico II, Naples, Italy; 8 Department of Neurosciences, Mental Health and Sensory Organs, Suicide Prevention Center, Sapienza University of Rome, Rome, Italy; 9 Department of Psychiatry, Hospital Papa Giovanni XXIII, Bergamo, Italy; 10 Psychiatric Unit, Department of Health Sciences, University Magna Graecia, Catanzaro, Italy; 11 Department of Neuroscience, Rehabilitation, Ophthalmology, Genetics, Maternal and Child Health, Section of Psychiatry, IRCCS Ospedale Policlinico San Martino, University of Genoa, Genoa, Italy; 12 Psychiatric Unit, Department of Clinical and Experimental Medicine, University of Foggia, Foggia, Italy; 13 Department of Basic Medical Sciences, Neuroscience and Sense Organs, University of Bari, Bari, Italy; 14 Department of Psychiatry, Fondazione Policlinico Universitario Agostino Gemelli IRCCS, Rome, Italy; 15 Department of Neuroscience, Section of Psychiatry, Università Cattolica del Sacro Cuore, Rome, Italy; 16 Department of Mental Health ASL, Bari, Italy; 17 Department of Psychiatry, University of Campania “L. Vanvitelli”, Naples, Italy; 18 Department of Mental Health and Addiction Services, ASST Spedali Civili, Brescia, Italy; 19 Department of Clinical and Experimental Sciences, University of Brescia, Brescia, Italy; 20 Department of Systems Medicine, University of Rome Tor Vergata, Rome, Italy; 21 Unit of Psychiatry and Clinical Psychology, Policlinico Tor Vergata Foundation, Rome, Italy; 22 Department of Psychiatry, University of Perugia, Perugia, Italy; 23 Department of Biomedical and Clinical Sciences “Luigi Sacco”, Psychiatry Unit 2, ASST-Fatebenefratelli-Sacco, via G.B.Grassi 74, 20157 Milan, Italy; 24 Department of Psychiatry and Behavioral Sciences, Stanford University, Stanford, CA USA; 25 CRC “Aldo Ravelli” for Neurotechnology and Experimental Brain Therapeutics, University of Milan, Milan, Italy

**Keywords:** Bipolar Disorder (BD), Suicidal attempts, Clinical features, Outcome

## Abstract

The purpose of the present study was to detect demographic and clinical factors associated with lifetime suicide attempts in Bipolar Disorder (BD). A total of 1673 bipolar patients from different psychiatric departments were compared according to the lifetime presence of suicide attempts on demographic/clinical variables. Owing to the large number of variables statistically related to the dependent variable (presence of suicide attempts) at the univariate analyses, preliminary multiple logistic regression analyses were realized. A final multivariable logistic regression was then performed, considering the presence of lifetime suicide attempts as the dependent variable and statistically significant demographic/clinical characteristics as independent variables. The final multivariable logistic regression analysis showed that an earlier age at first contact with psychiatric services (odds ratio [OR] = 0.97, *p* < 0.01), the presence of psychotic symptoms (OR = 1.56, *p* < 0.01) or hospitalizations (OR = 1.73, *p* < 0.01) in the last year, the attribution of symptoms to a psychiatric disorder (no versus yes: OR = 0.71, partly versus yes OR = 0.60, *p* < 0.01), and the administration of psychoeducation in the last year (OR = 1.49, *p* < 0.01) were all factors associated with lifetime suicide attempts in patients affected by BD. In addition, female patients resulted to have an increased association with life-long suicidal behavior compared to males (OR: 1.02, *p* < 0.01). Several clinical factors showed complex associations with lifetime suicide attempts in bipolar patients. These patients, therefore, require strict clinical monitoring for their predisposition to a less symptom stabilization. Future research will have to investigate the best management strategies to improve the prognosis of bipolar subjects presenting suicidal behavior.

## Introduction

Bipolar disorder (BD) is a widespread and severe psychiatric condition associated with reduced psychosocial functioning and a loss of life expectancy of about 10–20 years with respect to healthy general population, particularly due to suicide and cardiovascular diseases [1]. Different factors were identified as predictors of chronic course and poor outcome in BD, including among others delayed treatment [2], lifetime presence of psychotic symptoms [3, 4], rapid cycling [5–7], concomitant substance misuse [8], and psychiatric/medical comorbidity such as borderline personality disorder [9], obesity [10] or diabetes [11]. The identification of clinical predictors of illness severity allowed to a better management of BD, and to develop targeted prevention strategies [12]. However, BD continues to be considered one of the most challenging psychiatric illnesses by clinicians in light of the high risk of suicidal behavior associated with this condition [13]. In this context, lifetime attempted suicides represent a feature worth of investigation in bipolar patients for the associated unfavorable outcomes including frequent and severe mood episodes [14].

A first prospective large-sample study identified only past history of suicide attempts as main clinical predictor of suicide in BD [15]. This finding was confirmed by a Swedish registry-based investigation that indicated recent affective episodes and previous self-harm as clinical aspects related to high risk of attempted suicides in BD [16]. A subsequent article reported female gender, family history for mood disorders and psychiatric comorbidity with eating disorders as factors associated with suicide attempts in bipolar patients as well as previous suicidal behavior [17], particularly when performed with violent methods [18]. An American research group found female gender, BD-I subtype, psychiatric and substance use comorbidities, lifetime history of rapid cycling, and early age at onset as clinical predictors of self-harm in a large sample of bipolar patients [19]. Previously, gender, severity of depressive symptoms and family history for depression were also reported as factors associated with suicide attempts in a sample of early-onset young bipolar subjects [20]. An Indian study, focusing on a small sample of remitted bipolar type 1 patients, found that a family history of suicide attempts was the strongest predictor of future suicidal behavior (OR: 13.65, 95% confidence interval [CI]: 1.28–145.38) [21]. Finally, a 5-year follow-up study detected a higher risk of suicide attempts in bipolar subjects during a major depressive episode or a mixed one than in euthymia [22].

Preliminary Italian monocentric studies on small samples investigated the factors associated with suicidal behavior in BD. A first study found that a depressive polarity of first episode robustly predicted future suicide attempts in bipolar patients [23]. A subsequent investigation, focused on the clinical profile of Italian bipolar suicide attempters, reported that a number of variables such as more hospitalizations, psychiatric poly-comorbidity and depressive polarity of first episode were associated with a past history of suicide attempts [24]. The frequent depressive onset of bipolar suicide attempters may induce clinicians to prescribe antidepressants with consequent poor clinical stabilization and further risk of self-harm [25]. Finally, a very recent article reported sensitivity to weather and climate variations as important risk factors for lifetime suicide attempts in an Italian sample of bipolar patients [26].

The identification of factors associated with suicide attempts in BD can favor the implementation of prevention strategies and the prescription of a targeted pharmacotherapy [13]. In this sense, lithium should be the first choice in case of patients with suicidal ideation or attempts in the light of the well-established antisuicidal effects of this compound [27].

While studies exploring variables associated with suicide attempts in BD over the last decade have been conducted with different methodologies and limited samples, in this study, we sought to investigate, in a large multicentric sample of Italian bipolar patients, a wide series of socio-demographic and clinical features in relation with the presence of lifetime suicide attempts in BD.

## Methods

A total sample of 1673 patients with BD was enrolled from different Italian psychiatric clinics in the context of the RENDiBi project (National Epidemiological Research on Bipolar Disorder). The protocol was approved by the local Ethics Committees. Patients were diagnosed as affected by BD according to DSM-IV-TR (Diagnostic and Statistical Manual of Mental Disorders) criteria [28]. Diagnoses were performed by expert psychiatrists, who had regularly monitored the interviewed patients, and confirmed by the MINI International Neuropsychiatric Interview [29, 30]. Patients consecutively presenting at outpatient or inpatient clinics were selected for the purpose of the study.

Clinical information was obtained through a review of the clinical charts and clinical interviews with patients and available relatives. Two patients were not included in the analyses of this article for the lack of information about lifetime presence of suicide attempts. A detailed list of included Italian psychiatric clinics, as well as method of calculation of sample size, was previously reported [6]. Data were registered into an electronic central database (electronic Case Report Form: e-CRF developed by Mediolanum Cardio Research, Milan, Italy).

Collected data included the following socio-demographic and clinical variables:

- CLUSTER 1: socio-demographic variables: age, gender, education, employment, marital status (at least 1 lifetime marriage or partnership), living alone;
- CLUSTER 2: lifetime clinical variables: BD subtype, age at onset of BD, age at first psychopharmacological prescription (including benzodiazepines), age at first contact with psychiatric services, type of first psychiatric diagnosis, age at first psychiatric diagnosis, age at first diagnosis of BD, age at first prescription of mood stabilizer/atypical antipsychotic, polarity of first episode, predominant polarity, duration of illness (years), DUI (years), time between age at first psychopharmacological treatment and age at first treatment for BD (years), family history of psychiatric disorders (parents), lifetime presence of psychotic symptoms, lifetime number of manic episodes, lifetime number of hypomanic episodes, lifetime number of depressive episodes, lifetime number of mixed episodes, prevalent type of cycling, lifetime presence of medical comorbidity, lifetime presence of psychiatric comorbidity, lifetime more effective therapy;
- CLUSTER 3: clinical variables-last year of observation: presence of manic episodes, presence of depressive episodes, presence of hypomanic episodes, presence of mixed episodes, presence of psychotic symptoms, comorbidity with substance misuse, presence of hospitalizations, presence of insight, attribution of symptoms to a psychiatric disorder, treatment adherence, number of visits, time between visits for euthymic patients (days),current administration of psychotherapy, administration of psychoeducational interventions (according to Colom’s model) [31].

Duration of untreated illness was considered as the time elapsing between first episode of BD and the prescription of a proper pharmacological treatment (mood stabilizer or atypical antipsychotic with stabilizing effects) [2]. Predominant polarity was calculated according to the Barcelona proposal, defined as at least two-thirds (2/3) of the total number of past episodes being from the same polarity [32]. The presence of psychotic symptoms refers to all types of major mood episodes (manic, mixed or depressive ones). The variable “attribution of symptoms to a psychiatric disorder” refers to patients denying fully or partially that their symptoms belong to a psychiatric condition, differentiating from “insight” that was defined as the awareness to have symptoms.

Suicide attempt was defined as self-harm combined with the intent to die. Self-harm without intent was not taken into account. Similarly to the other variables, the information about suicide attempts was obtained through a review of the clinical charts and clinical interviews with patients and available relatives and registered into the e-CRF.

Exclusion criteria included: (1) patients who had not been screened in the last 12 months making it impossible to collect data for the last year of observation) and (2) patients whose clinical information was incomplete.

Descriptive analyses of the total sample were performed. The total sample was then divided in two groups according to the presence of lifetime suicide attempts. The two groups were compared for the above mentioned variables by *t* tests for quantitative variables and Chi square tests for qualitative ones.

Owing to the large number of variables statistically related to the dependent variable (the presence of lifetime suicide attempts) at the univariate analyses, preliminary multiple logistic regression analyses (for each of the above mentioned clusters) were performed including only statistically significant variables. In the model, we included also age, despite being non-significant in the univariate analyses, being the younger and the older individuals more prone to impulsivity and suicidal behaviors [33]. Finally, statistically significant variables from these final models were inserted in a new global multivariable logistic regression model (adjusted for gender) to obtain the variables independently associated with the presence of lifetime suicide attempts. The choice to adjust the final model for gender was due to the fact that another analysis on the sample showed demographic and clinical differences according to this variable [34].

The selection of the variables was done according to a backward procedure; the goodness of fitting was assessed by the Hosmer–Lemeshow test.

The level of statistical significance was set at *p* ≤ 0.05. Statistical analyses were performed by SAS^®^ 9.2 version.

## Results

The total sample included 1673 patients: 714 males (42.7%) and 959 females (57.3%). Four hundred and forty patients (26.3% of the total sample) reported at least one lifetime suicide attempts. At the moment of the assessment, 242 subjects were experiencing a major depressive episode (14.4%), 198 (11.9%) mania, 26 (1.6%) hypomania, 150 (9.0%) a mixed episode, and 1057 (63.1%) were euthymic.

Descriptive analyses of the total sample and groups divided according to the lifetime presence of suicide attempts are reported in Tables 1, 2, 3.

**Table 1:** Socio-demographic variables of the total sample and of the two groups obtained according to the presence of lifetime suicide attempts

Variables		Total sample *N* = 1673	Absence of lifetime suicide attempts *N* = 1233 (73.7%)	Presence of lifetime suicide attempts *N* = 440 (26.3%)	*p*
Gender Missing: *n* = 0	Male	714 (42.7%)	553 (44.9%)	161 (36.6%)	** < 0.01**
	Female	959 (57.3%)	680 (55.1%)	279 (63.4%)	
Education (years) Missing: *n* = 5	< 13	603 (36.1%)	436 (35.4%)	167 (38.1%)	**0.02**
	< 16	749 (44.9%)	541 (44.0%)	208 (47.5%)	
	≥ 16	316 (19.0%)	253 (20.6%)	63 (14.4%)	
Employed missing: *n* = 3	Yes	1264 (75.7%)	943 (76.5%)	321 (73.3%)	0.17
	No	406 (24.3%)	289 (23.5%)	117 (26.7%)	
Marriage or partnership missing: *n* = 2	≥ 1	1138 (68.1%)	820 (66.6%)	318 (72.4%)	**0.02**
	Never	533 (31.9%)	412 (33.4%)	121 (27.6%)	
Living alone missing: *n* = 2	Yes	287 (17.2%)	204 (16.6%)	83 (18.9%)	0.26
	No	1384 (82.8%)	1028 (83.4%)	356 (81.1%)	
Age missing: *n* = 0		48.62 (± 13.44)	48.44 (± 13.88)	49.11 (± 12.13)	0.37

Standard deviations for the variable “age” and percentages for qualitative variables are reported into brackets

In bold statistically significant p resulting from *χ*^2^ and from unpaired Student’s *t* test for the variable “age”

**Table 2:** Clinical variables of the total sample and of the two groups obtained according to the presence of lifetime suicide attempts

Variables		Total sample *N* = 1673	Absence of lifetime suicide attempts *N* = 1233 (73.7%)	Presence of lifetime suicide attempts *N* = 440 (26.3%)	*p*
Age at onset of BD					
Missing: *n* = 70		31.50 (± 12.02)	32.03 (± 12.31)	29.99 (± 11.20)	< 0.01
Age at first pharmacological prescription (including BZP)					
Missing: *n* = 56		30.87 (± 11.77)	31.58 (± 12.31)	28.86 (± 10.24)	< 0.01
Age at first contact with psychiatric services					
Missing: *n* = 56		31.23 (± 12.08)	31.96 (± 12.53)	29.16 (± 10.80)	< 0.01
Age at first psychiatric diagnosis					
Missing: *n* = 51		30.62 (± 11.81)	31.31 (± 12.29)	28.64 (± 10.46)	< 0.01
First psychiatric diagnosis missing: *n* = 47	MDD	713 (43.9%)	520 (43.3%)	193 (45.5%)	< 0.01
	BD	532 (32.7%)	412 (34.3%)	120 (28.3%)	
	Eating disorders	20 (1.2%)	11 (0.9%)	9 (2.1%)	
	Anxiety disorders	147 (9.0%)	117 (9.7%)	30 (7.1%)	
	Substance abuse	32 (2.0%)	25 (2.1%)	7 (1.7%)	
	Other disorders	182 (11.2%)	117 (9.7%)	65 (15.3%)	
Age at first diagnosis of BD missing: *n* = 61		37.54 (± 13.37)	37.96 (± 13.66)	36.37 (± 12.54)	**0.03**
Age at first Mood Stabilizer/Atypical Antipsychotic missing: *n* = 72		37.48 (± 13.35)	37.89 (± 13.64)	36.33 (± 12.54)	**0.03**
Duration of illness (years) Missing: *n* = 79		17.22 (± 12.44)	16.52 (± 12.56)	19.16 (± 12.12)	** < 0.01**
Time between the first treatment for BD and first psychiatric treatment		6.72 (± 9.26)	6.45 (± 9.06)	7.49 (± 9.83)	0.06
Duration of untreated illness (years) missing: *n* = 97		5.96 (± 9.65)	5.84 (± 9.56)	6.29 (± 9.91)	0.36
Polarity of first episode missing: *n* = 43	Depressive	911 (55.9%)	636 (52.7%)	275 (64.9%)	** < 0.01**
	Hypomanic/Manic	577 (35.4%)	457 (37.9%)	120 (28.3%)	
	Unidentifiable (Mixed episode)	142 (8.7%)	113 (9.4%)	29 (6.8%)	
Lifetime number of manic episodes Missing: *n* = 21	0	633 (38.3%)	478 (39.2%)	155 (35.7%)	0.13
	1–2	444 (26.9%)	326 (26.8%)	118 (27.2%)	
	3–5	343 (20.8%)	257 (21.1%)	86 (19.8%)	
	≥ 6	232 (14.0%)	157 (12.9%)	75 (17.3%)	
Lifetime number of mixed episodes missing: *n* = 20	0	1040 (63.0%)	823 (67.4%)	217 (50.2%)	** < 0.01**
	1–2	333 (20.0%)	241 (19.7%)	92 (21.3%)	
	3–5	183 (11.1%)	107 (8.8%)	76 (17.6%)	
	≥ 6	97 (5.9%)	50 (4.1%)	47 (10.9%)	
Lifetime number of hypomanic episodes missing: *n* = 25	0	750 (45.5%)	554 (45.4%)	196 (45.7%)	0.55
	1–2	410 (24.9%)	313 (25.7%)	97 (22.6%)	
	3–5	259 (15.7%)	186 (15.3%)	73 (17.0%)	
	≥ 6	229 (13.9%)	166 (13.6%)	63 (14.7%)	
Lifetime number of depressive episodes missing: *n* = 9	0	156 (9.4%)	127 (10.4%)	29 (6.6%)	** < 0.01**
	1–2	452 (27.1%)	360 (29.3%)	92 (21.1%)	
	3–5	464 (27.9%)	342 (27.9%)	122 (27.9%)	
	≥ 6	592 (35.6%)	398 (32.4%)	194 (44.4%)	
Family history for psychiatric disorders (father) missing: *n* = 69	None	1261 (78.6%)	937 (79.3%)	324 (76.8%)	**0.01**
	MDD	83 (5.2%)	68 (5.7%)	15 (3.6%)	
	BD	109 (6.8%)	79 (6.7%)	30 (7.1%)	
	Anxiety disorders	47 (3.0%)	35 (3.0%)	12 (2.8%)	
	Other psychiatric disorders	104 (6.4%)	63 (5.3%)	41 (9.7%)	
Family history for psychiatric disorders (mother) Missing: *n* = 68	None	1034 (64.4%)	773 (65.3%)	261 (61.8%)	0.36
	MDD	276 (17.2%)	198 (16.7%)	78 (18.5%)	
	BD	140 (8.7%)	99 (8.4%)	41 (9.7%)	
	Anxiety disorders	107 (6.7%)	82 (7.0%)	25 (6.0%)	
	Other psychiatric disorders	48 (3.0%)	31 (2.6%)	17 (4.0%)	
BD type Missing: *n* = 175	1	963 (64.3%)	687 (61.9%)	276 (71.1%)	** < 0.01**
	2	535 (35.7%)	423 (38.1%)	112 (28.9%)	
Lifetime psychotic symptoms Missing: *n* = 2	No	891 (53.3%)	696 (56.5%)	195 (44.3%)	** < 0.01**
	Yes	780 (46.7%)	535 (43.5%)	245 (55.7%)	
Lifetime psychiatric comorbidity Missing: *n* = 3	No	884 (53.0%)	671 (54.6%)	213 (48.4%)	**0.03**
	Yes	786 (47.0%)	559 (45.4%)	227 (51.6%)	
Lifetime more effective therapy missing: *n* = 119	Mood Stabilizers	1022 (65.8%)	770 (67.1%)	252 (61.9%)	0.16
	Antidepressants	139 (8.9%)	98 (8.6%)	41 (10.1%)	
	Antipsychotics	393 (25.3%)	279 (24.3%)	114 (28.0%)	
Lifetime medical comorbidity missing: *n* = 46	No	1187 (73.0%)	880 (73.5%)	307 (71.6%)	0.45
	Yes	440 (27.0%)	318 (26.5%)	122 (28.4%)	
Prevalent type of cycling missing: *n* = 7	Manic/Depressive	347 (20.8%)	263 (21.4%)	84 (19.2%)	0.59
	Depressive/Manic	387 (23.2%)	281 (22.9%)	106 (24.2%)	
	Other	932 (56.0%)	684 (55.7%)	248 (56.6%)	
Predominant polarity missing: *n* = 3	Depressive	791 (47.4%)	555 (45.1%)	236 (53.9%)	** < 0.01**
	Hypomanic/Manic	438 (26.2%)	354 (28.7%)	84 (19.2%)	
	Unidentifiable	441 (26.4%)	323 (26.2%)	118 (26.9%)	

Standard deviations for quantitative variables and percentages for qualitative variables are reported into brackets

In bold statistically significant *p* resulting from *χ*^2^ or unpaired Student’s *t* tests

*BZP* Benzodiazepines, *BD* Bipolar Disorder, *MDD* Major Depressive Disorder

**Table 3:** Clinical variables of the total sample and of the two groups obtained according to the presence of lifetime suicide attempts (last year of observation)

Variables		Total Sample *N* = 1673	Absence of Lifetime Suicide Attempts *N* = 1233 (73.7%)	Presence of Lifetime Suicide Attempts *N* = 440 (26.3%)	*p*
Presence of psychotic symptoms missing: *n* = 4	No	1262 (75.6%)	958 (77.8%)	304 (69.3%)	** < 0.01**
	Yes	407 (24.4%)	272 (22.1%)	135 (30.7%)	
Presence of hospitalizations missing: *n* = 0	No	987 (59.0%)	777 (63.0%)	210 (47.7%)	** < 0.01**
	Yes	686 (41.0%)	456 (37.0%)	230 (52.3%)	
Presence of depressive episodes missing: *n* = 98	No	865 (54.9%)	666 (57.2%)	199 (48.4%)	** < 0.01**
	Yes	710 (45.1%)	498 (42.8%)	212 (51.6%)	
Presence of mixed episodes missing: *n* = 97	No	1317 (83.6%)	990 (85.0%)	327 (79.4%)	** < 0.01**
	Yes	259 (16.4%)	174 (15.0%)	85 (20.6%)	
Presence of hypomanic episodes missing: *n* = 116	No	1253 (80.5%)	920 (80.1%)	333 (81.4%)	0.58
	Yes	304 (19.5%)	228 (19.9%)	76 (18.6%)	
Presence of manic episodes missing: *n* = 91	No	1266 (80%)	931 (79.7%)	335 (80.9%)	0.60
	Yes	316 (20.0%)	237 (20.3%)	79 (19.1%)	
Insight missing: *n* = 2	No	78 (4.7%)	60 (4.9%)	18 (4.1%)	**0.05**
	Yes	1165 (69.7)	839 (68.1%)	326 (74.3%)	
	Partial	428 (25.6%)	333 (27.0%)	95 (21.6%)	
Attribution of symptoms to a psychiatric disorder missing: *n* = 3	No	131 (7.8%)	99 (8.0%)	32 (7.3%)	** < 0.01**
	Yes	1064 (63.7%)	758 (61.6%)	306 (69.7%)	
	Partial	475 (28.5%)	374 (30.4%)	101 (23.0%)	
Treatment adherence missing: *n* = 7	No	124 (7.4%)	89 (7.2%)	35 (8.0%)	0.81
	Yes	1156 (69.4%)	850 (69.3%)	306 (69.7%)	
	Partial	386 (23.2%)	288 (23.5%)	98 (22.3%)	
Number of visits missing: *n* = 103		9.67 (± 9.07)	9.22 (± 8.62)	10.95 (± 11.94)	** < 0.01**
Number of days between visits when patients are in euthymia missing: *n* = 178		46.63 (± 40.28)	47.65 (± 40.04)	43.72 (± 40.94)	0.10
Psychoeducation missing: *n* = 8	No	1394 (83.7%)	1044 (85.1%)	350 (79.9%)	**0.01**
	Yes	271 (16.3%)	183 (14.9%)	88 (20.1%)	
Substance misuse missing: *n* = 1	No	1471 (88.0%)	1083 (87.9%)	388 (88.2%)	0.88
	Yes	201 (12.0%)	149 (12.1%)	52 (11.2%)	
Current psychotherapy missing: *n* = 5	No	1423 (85.3%)	1058 (86.0%)	365 (83.3%)	0.17
	Yes	245 (14.7%)	172 (14.0%)	73 (16.7%)	

Standard deviations for quantitative variables and percentages for qualitative variables are reported into brackets

In bold statistically significant results from *χ*^2^ or unpaired Student’s *t* tests

There were no significant differences between groups identified by the lifetime presence of suicide attempts in terms of age (*p* = 0.37), employment (*p* = 0.17) and living alone status (*p* = 0.26), time between the first treatment for BD and first psychiatric treatment (p = 0.06), duration of untreated illness (*p* = 0.36), lifetime number of manic episodes (*p* = 0.13), lifetime number of hypomanic episodes (*p* = 0.55), family history for psychiatric disorders (mothers) (*p* = 0.36), type of lifetime more effective therapy (*p* = 0.16), lifetime medical comorbidity (*p* = 0.45), prevalent type of cycling (*p* = 0.59), presence of hypomanic (*p* = 0.58) and manic (*p* = 0.60) episodes in the last year of observation, treatment adherence (*p* = 0.81), number of days between visits when patients are euthymic (*p* = 0.10), substance misuse in the last year of observation (*p* = 0.88), current administration of psychotherapy (*p* = 0.17).

On the other hand, subjects with lifetime suicide attempts resulted: to be more frequently women (*p* < 0.01), less educated (*p* = 0.02), to have had at least one lifetime marriage or partnership (*p* = 0.02), to have an earlier age at onset (*p* < 0.01), to have an earlier age at first pharmacological prescription (*p* < 0.01), to have an earlier age at first contact with psychiatric services (*p* < 0.01), to have an earlier age at first psychiatric diagnosis (*p* < 0.01), to have an earlier age at first diagnosis of BD (*p* = 0.03), to have an earlier age at first mood stabilizer or atypical antipsychotic prescription (*p* = 0.03), to have received more frequently a first diagnosis different from BD (in most of cases of a Major Depressive Disorder) (*p* < 0.01), to have a longer duration of illness (*p* < 0.01), to more frequently show depressive polarity of first episode (*p* < 0.01) and depressive predominant polarity (p < 0.01), to present a higher lifetime number of depressive episodes (*p* < 0.01), to present more lifetime mixed episodes (*p* < 0.01), to show a more frequent paternal family history of BD (*p* = 0.01), to have a diagnosis of BD type 1 (p < 0.01), to present more frequent lifetime psychotic symptoms (*p* < 0.01) and psychiatric comorbidity (*p* = 0.03), to have a higher number of depressive (*p* < 0.01) and mixed episodes (*p* < 0.01) in the last year, to have more insight (*p* = 0.05), to more frequently attribute symptoms to a psychiatric disorder (*p* < 0.01), to have more frequently received psychoeducation in the last year (*p* = 0.01), to have more hospitalizations in the last year (*p* < 0.01), to present more psychotic symptoms in the last year (*p* < 0.01), to have had more visits in the last year (*p* < 0.01).

The results of the goodness-of-fit test (Hosmer and Lemeshow Test: *χ*^2^ = 5.14, df = 8, *p* = 0.74) showed that final multivariable logistic regression model including socio-demographic/clinical variables as possible associated factors of the lifetime presence of suicide attempts (adjusted for gender) was reliable. Of note, bipolar patients with versus without lifetime presence of suicide attempts resulted to be older (odds ratio-OR = 1.02, *p* < 0.01), to have an earlier age at first contact with psychiatric services (OR = 0.97, *p* < 0.01), to present more frequently psychotic symptoms in the last year (OR = 1.56, *p* < 0.01), to present more frequently hospitalizations in the last year (OR = 1.73, *p* < 0.01), to attribute more frequently the symptoms to a psychiatric disorder (no versus yes: OR = 0.71, partly versus yes OR = 0.60, *p* < 0.01), to have received more frequently psychoeducation in the last year (OR = 1.49, *p* < 0.01), and finally to have had more visits at a borderline statistical significance (OR = 1.01, *p* = 0.06) (Table 4).

**Table 4:** Summary of the statistics for the best-fit multivariable logistic regression model applied

Variables	Categories	Odds Ratio	95%CI	*p*
Age	NA	1.02	1.01–1.03	** < 0.01**
Gender	Female vs male	1.20	0.95–1.53	0.15
Age at first contact with psychiatric services	NA	0.97	0.96–0.99	** < 0.01**
Presence of psychotic symptoms in the last year	Yes vs. no	1.45	1.01–1.92	** < 0.01**
Presence of hospitalizations in the last year	Yes vs. no	1.73	1.35–2.22	** < 0.01**
Attribution of symptoms to a psychiatric disorder	No vs. yes	0.71	0.44–1.17	** < 0.01**
	Partial vs. yes	0.60	0.45–0.80	
Administration of psychoeducation in the last year	No vs. yes	0.67	0.50–0.90	** < 0.01**
Number of visits in the last year	NA	1.01	0.99–1.02	0.06

In this analysis, the dependent variable was lifetime presence of suicidal attempts. With regard to age and number of visits, the odds ratio means the increase of the association for each unit of year or visit increase. However, OR is 1.09 (95%CI: 1.04–1.15), 1.19 (95% CI: 1.07–1.32), 1.30 (95% CI: 1.14–1.52) for 5, 10 and 15 years increase, respectively. Furthermore, OR is 1.02 (95% CI: 0.99–1.05), 1.04 (95% CI: 0.99–1.07), 1.05 (95% CI: 0.99–1.10) for 2, 3 and 4 visit increase, respectively

Hosmer–Lemeshow test: *χ*^2^ = 5.14, df = 8, *p* = 0.74

*Vs* = versus; *NA* = not applicable; *95% CI* = 95% Confidence Interval

In bold statistically significant *p* values

In addition, removing from the above multivariable model the variables psychoeducation in the last year, number of hospitalizations, and number of visits, in the light of their not unique temporal relationship with the lifetime presence of suicide attempts leading to a controversial interpretation of their role, bipolar patients with versus without lifetime presence of suicide attempts resulted to be older (OR = 1.02, *p* < 0.01), to have an earlier age at first contact with psychiatric services (OR = 0.97, *p* < 0.01), to present more frequently psychotic symptoms in the last year (OR = 1.84, *p* < 0.01), to attribute more frequently the symptoms to a psychiatric disorders (no versus yes: OR = 0.65, partly versus yes OR = 0.60, *p* < 0.01). The results of the goodness-of-fit test (Hosmer and LemeshowTest: *χ*^2^ = 3.52, df = 8, *p* = 0.59) showed that this multivariable logistic regression model, adjusted for gender (female versus male OR = 1.23, *p* = 0.08) was reliable.

Finally, if in the multivariable logistic model we insert only the variables that are supposed to precede or to be independent from the first lifetime suicide attempt, the ORs (95% confidence interval-CI) result to be 1.28 (CI:1.02–1.61, *p* = 0.03) for gender (female versus male) and 0.97 (CI: 0.96–0.98, *p* < 0.01) for each unit increase of age at first contact with psychiatric services. Therefore, an earlier age at first contact with psychiatric services is confirmed as a factor associated with lifetime suicide attempts, but in this case also female gender resulted to be significantly related with suicidal behaviors. The above estimates and significance remain practically unchanged if we add in the model the variable age (1.02 for each unit increase: CI:1.01–1.03, *p* < 0.01) for having the odds ratios adjusted for.

## Discussion

To authors’ knowledge, this is the first multicenter study conducted in Italy with a large sample of bipolar patients, with the aim to assess a large set of variables in relation to lifetime suicide attempts. About 26% of the total sample reported at least one lifetime suicidal attempts. This figure is lower with respect to that reported by other studies [35] and this aspect can be explained by differences in treatment in our patients in comparison to other reported case series and by the fact that some of our patients were followed in tertiary care clinics.

Lifetime presence of suicide attempts resulted to be associated with a number of demographic and clinical variables, including a lower level of education, the presence of a lifetime marriage/partnership, female gender, an earlier age at onset, a first psychiatric misdiagnosis with a Major Depressive Disorder, a longer duration of illness, a depressive polarity of first mood episode, lifetime number of mixed/depressive episodes, paternal family history of BD, BD type 1, lifetime psychiatric comorbidity, depressive predominant polarity, recent depressive/mixed episodes, presence of insight. In addition, even a more robust association, as shown by the final multivariable logistic regression model, was found between the lifetime presence of suicidal behavior in bipolar patients and an earlier age at first contact with psychiatric services, last year presence of hospitalizations and psychotic symptoms, recent administration of psychoeducation, attribution of symptoms to a psychiatric disorder and number of visits in the last year at a borderline statistically significant level. Finally, the multivariable logistic regression model including only the factors supposed to precede or to be independent from the first lifetime suicide attempt show a relation of an earlier age at first contact with psychiatric services and female gender with suicidal behaviors. While some of these findings confirm results of previous reports, other results are mostly unprecedented.

The level of education has been poorly investigated in terms of suicidal behaviors in patients with BD. A recent article reported that a higher level of education was more frequent in subjects with BD type 2 [36] that in turn, accordingly to our data, seemed be less associated with a lifetime risk of suicide attempts. With regard to marital status, our findings seem to contradict previous literature revealing that people who are single or divorced present a greater vulnerability to suicide [7]. These opposite results can be explained by the different way to classify marital status in our dataset (considering lifetime marriages/partnerships) than the previous published articles about this topic [13]. Of note, the variable “lifetime marriages” includes separated or divorced individuals who might have experienced more unstable relationships. Our data confirmed that female gender remains one of the factors associated with lifetime suicide attempts in BD in agreement with several previous studies [17, 19, 20].

With regard to clinical variables, the results of the present study confirm the findings from previous studies, specifically showing that suicidal behavior in BD is predicted by a number of variables associated with a more severe course of illness, such as an early age at onset [37], a longer duration of illness [38], family history of BD [17] or the presence of psychiatric comorbidity [17, 19, 37].

As confirmed by our data in agreement with previous literature, depressive symptoms, namely recent/lifetime depressive episodes [16] and predominant depressive polarity, seem to have a prominent role in conferring vulnerability to suicide attempts in bipolar patients [15, 20]. Similarly, the presence of mixed symptoms, likely associated with higher levels of impulsivity, was found to be predictive of suicidal behavior in BD in several reports [22] as well as in our study. As highlighted in previous studies by our [2, 3] and other research groups [39], a depressive polarity of the first episode in bipolar patients with higher risk of suicide attempts [23] may favor misdiagnosis of these subjects who, treated with antidepressant mono-therapy, can show a worse prognosis in terms of inadequate clinical stabilization, greater impulsivity and occurrence of suicide attempts [25, 40].

Data about the association of bipolar subtype and presence of suicidal behavior are more difficult to interpret with studies reporting a relation with BD type 2 [18] and others that, in agreement with our findings, detected more propensity to suicide attempts in bipolar 1 patients [19]. This contradiction is not totally surprising as other factors such as depressive symptoms, more prevalent in the longitudinal course of BD type 2, may contribute to the vulnerability to suicidal behavior. Nonetheless, also bipolar type 1 patients spend most of their illness in depression that is one of the main predictors of suicidal attempts [41]. Ultimately, complex associations of different variables concur to confer a higher risk of suicide attempts across bipolar subtypes, including also cultural and geographical aspects [42] and specific patterns of comorbidity [43].

Of note, our results detected a larger amount of insight in patients with lifetime suicide attempts contradicting the results of previous research articles [44]. This finding can be explained by the fact that our patients with suicide attempts also received more psychoeducation than those without previous suicide attempts. Another possible reason is that suicide attempts occur more frequently in patients with depressive polarity or with recent depressive episodes that usually are characterized by more insight with respect to mania [45]. Finally, a relatively recent study [46] found that not only the lack, but also the change of insight is associated with suicidal behaviors in patients affected by severe mental disorders such as BD where the different mood phases are characterized by variable insight. In support of the mentioned hypotheses, recent research highlighted that insight coupled with depression may be associated to an increased suicidal risk in patients affected by severe mental conditions [47].

Finally, the results of the present study clearly indicate that patients with lifetime suicide attempts present less clinical stability in terms of more recent hospitalizations, psychotic symptoms and number of visits, therefore requiring a more strict follow-up and an integrated therapy (e.g., with pharmacotherapy and psychoeducation) [48]. Future research will have to investigate the best management strategies to improve the prognosis of bipolar subjects presenting suicidal behavior, eventually differentiating between single versus multiple suicide attempters.

The abovementioned results need to be interpreted in light of the following limitations:

1. patients were treated with different drugs which might have influenced some clinical features (e.g., the prescription of first generation antipsychotics during mania favoring the switch to depression);
2. the different settings of care (in several Italian regions) may have influenced the clinical features of the sample (e.g., the availability of psychoeducation or of targeted day hospital units for mood disorders);
3. even though some of the patients were selected in community primary care services, others have been treated in tertiary academic care units, with a potentially differential influence of the setting over some clinical features (e.g., more severe patients are usually treated in secondary or tertiary care units);
4. some data were collected retrospectively (e.g., number of mood episodes, past attempted suicides) so that they might have not been always as accurate as in controlled studies;
5. the lack of a follow-up period due to the cross-sectional nature of the study that limits any causal inference;
6. the impossibility to perform an analysis considering single versus multiple suicide attempters.

Finally, the findings presented in this article have not to be interpreted in the terms of causal inference, but only as the associations between the independent variables and the event, given for granted their possible role as confounders, as it should be highlighted for all the multivariable models fitted in the biomedical observational research.
